# *In Vitro* Evaluation of the Effects of *Eurycoma longifolia* Extract on CYP-Mediated Drug Metabolism

**DOI:** 10.1155/2015/631329

**Published:** 2015-07-09

**Authors:** Young Min Han, In Sook Kim, Shaheed Ur Rehman, Kevin Choe, Hye Hyun Yoo

**Affiliations:** Institute of Pharmaceutical Science and Technology and College of Pharmacy, Hanyang University, Ansan, Gyeonggi-do 426-791, Republic of Korea

## Abstract

*Eurycoma longifolia* (Simaroubaceae) is a popular folk medicine that has traditionally been used in Southeast Asia as an antimalarial, aphrodisiac, antidiabetic, and antimicrobial and in antipyretic remedies. This study evaluates the effects of *Eurycoma longifolia* extract on cytochrome P450 (CYP) enzyme-mediated drug metabolism to predict the potential for herb-drug interactions. Methanolic extract of *E. longifolia * root was tested at concentrations of 1, 3, 10, 30, 100, 300, and 1000 *µ*g/mL in human liver microsomes or individual recombinant CYP isozymes. The CYP inhibitory activity was measured using the cocktail probe assay based on liquid chromatography-tandem mass spectrometry. *E. longifolia* showed weak, concentration-dependent inhibition of CYP1A2, CYP2A6, and CYP2C19. The inhibitory effects on these CYP isozymes were further tested using individual recombinant CYP isozymes, showing IC_50_ values of 324.9, 797.1, and 562.9 *μ*g/mL, respectively. In conclusion, *E. longifolia* slightly inhibited the metabolic activities of CYP1A2, CYP2A6, and CYP2C19 but this issue requires careful attention in taking herbal medicines or dietary supplements containing *E. longifolia* extracts.

## 1. Introduction

*Eurycoma longifolia* jack (Simaroubaceae) is used as a herbal medicine and functional food in many Southeast Asian countries, including Indonesia, Malaysia, Thailand, Vietnam, and Laos [1].* E. longifolia* has been reported to contain various types of secondary metabolites such as quassinoids [2], canthinones [3], squalene derivatives [4], and *β*-carboline alkaloids [5]. Root extracts of* E. longifolia* have long been used to treat fatigue [6], malaria [7], inflammation [8], and persistent fever [9]. In addition, it is known to have ergogenic effect [10] and to improve erectile dysfunction [11]. Due to these pharmacological effects,* E. longifolia* extracts are commonly used as dietary supplements or alternative medicine.

The use of traditional herbal therapies is becoming more common. Recently, the World Health Organization estimated that 80% of people worldwide rely on herbal medicines for some part of their primary health care [12]. Accordingly, patients may take herbal medicines in combination with prescription or conventional medications without employing appropriate precautions. However, such coadministration of herbal remedies and conventional medication may lead to herb-drug interactions, resulting in unexpected adverse effects, altered drug response, or lack of drug efficacy.

Herb-drug interactions may interfere with various pharmacokinetic processes: herbs have been reported to alter drug absorption, induction and inhibition of metabolic enzymes, and drug excretion [13]. In particular, modulation of drug metabolic enzymes such as cytochrome P450 (CYP) by herbs is well recognized as a primary cause of herb-drug interactions. For example, herbal medicines may inhibit CYP enzyme activity, thereby altering the pharmacokinetics of the coadministered drug that is the substrate of the CYP enzyme; this may cause toxicity due to the increased plasma concentration level of the drug. Therefore, evaluation of herb-drug interactions associated with drug-metabolizing enzymes is necessary to ensure the safe use of herbal products.

*E. longifolia* extract should be evaluated for possible herb-drug interactions, as it is likely coadministered with conventional drugs. However, to our knowledge, there has been no previous study that examined the effects of* E. longifolia* on CYP enzyme activities. Therefore, this study examined the inhibitory effects of* E. longifolia* extract on CYP450-mediated drug metabolism, using human liver microsomes and individual recombinant CYP isozymes.

## 2. Materials and Methods

### 2.1. Chemicals and Reagents

The methanolic extract of* E. longifolia* roots was provided by the Institute of Marine Biochemistry, Vietnam Academy of Science and Technology (Hanoi, Vietnam). Pooled human liver microsomes and recombinant CYP1A2, CYP2A6, and CYP2C19 isozymes were purchased from BD Gentest (Woburn, MA, USA). Glucose-6-phosphate, *β*-NADP^+^, glucose-6-phosphate dehydrogenase, phenacetin, coumarin, diclofenac, mephenytoin, dextromethorphan, midazolam, ketoconazole, and terfenadine were obtained from Sigma Chemical Co. (Saint Louis, MO, USA). All other solvents used were of HPLC grade and were purchased from J. T. Baker (Phillipsburg, NJ, USA). Distilled water was prepared using a Milli-Q purification system (Millipore, Billerica, MA, USA).

### 2.2. HPLC Analysis of* E. longifolia* Extracts

**Figure 1:**
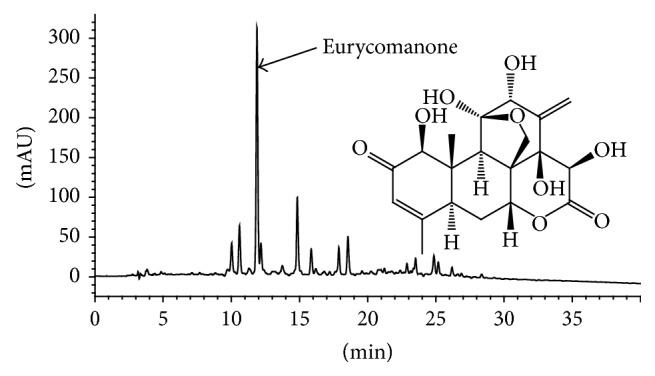
Representative HPLC chromatogram of* Eurycoma longifolia* extracts. The extract of* E. longifolia* (1 mg/mL) was analyzed by HPLC with a C18 column. The UV detection wavelength was set at 244 nm. The retention time of eurycomanone was 13.4 min and the resulting content was calculated as 1.3 ± 0.1% of* E. longifolia* extract (*n* = 3).

The extract of* E. longifolia* was dissolved in distilled water (1 mg/mL). The sample was filtered through a 0.22 *μ*m membrane filter, and a 10 *μ*L aliquot of the sample was injected into an HPLC system (1260 infinity HPLC system; Agilent Technologies) equipped with an ultraviolet (UV) detector. The analytical column (Capcell Pak C18: 4.6 mm × 250 mm, 5 *μ*m; Shiseido) was maintained at 30°C. The mobile phase consisted of 0.1% formic acid (solvent A) and 90% acetonitrile with 0.1% formic acid (solvent B). The flow rate was set at 1 mL/min. The gradient elution mode was used; the concentration of solvent B was gradually increased from 5% to 25% for 30 min, further increased to 90% for 5 min, maintained at 90% for 1 min, decreased to the initial condition for 1 min, and maintained for reequilibrium. The injection volume was 10 *μ*L, and the UV detection wavelength was set at 244 nm. In the HPLC chromatogram (Figure 1), eurycomanone (retention time 13.4 min) was found to be a principle constituent (content 1.3 ± 0.1%, *n* = 3) of* E. longifolia* extract.

### 2.3. CYP Inhibition Assay

The reaction mixtures consisted of 0.5 mg/mL human liver microsomes; various concentrations of* E. longifolia* extract (1, 3, 10, 30, 100, 300, and 1000 *μ*g/mL in distilled water); an NADPH-generating system (NGS) containing 0.1 M glucose-6-phosphate, 10 mg/mL *β*-NADP^+^, and 1.0 U/mL glucose-6-phosphate dehydrogenase, and a substrate mixture (40 *μ*M phenacetin, 2.5 *μ*M coumarin, 5 *μ*M dextromethorphan, 10 *μ*M diclofenac, 160 *μ*M mephenytoin, 10 *μ*M paclitaxel, and 2.5 *μ*M midazolam) in 200 *μ*L of 0.05 M potassium phosphate buffer (pH 7.4). The reaction mixture was preincubated at 37°C for 5 min without NGS and then further incubated for 30 min with NGS in a water bath. Ketoconazole (5 *μ*M), furafylline (10 *μ*M), methoxsalen (10 *μ*M), sulfaphenazole (50 *μ*M), ticlopidine (20 *μ*M), quercetin (30 *μ*M), and quinidine (50 *μ*M) were tested as positive controls. After the incubation, the reaction was stopped by adding 400 *μ*L of 0.1% acetic acid containing internal standard (0.16 *μ*M terfenadine). For the CYP inhibition assay using each CYP isozyme, 12.5 pmol isozyme (CYP 1A2, 2A6, and 2C19) instead of human liver microsomes and the corresponding specific substrate (40 *μ*M phenacetin, 2.5 *μ*M coumarin, and 160 *μ*M mephenytoin, resp.) were added to the reaction mixture and the rest of the procedure was performed as described above.

### 2.4. Sample Preparation

The incubation mixtures were passed through activated Sep-Pak C18 cartridges (96-well OASIS HLB Extraction Cartridge, Waters, Milford, MA, USA). The cartridges were eluted with methanol (1 mL) and 0.1% acetic acid (2 mL). After sample loading, the cartridges were washed twice with 1 mL of 0.1% acetic acid and eluted with 1 mL of methanol. The eluate was evaporated under nitrogen gas, the residue was reconstituted in 100 *μ*L of the mobile phase (0.1% formic acid in an 85 : 15 mixture of water : acetonitrile), and a 5 *μ*L aliquot was injected into the liquid chromatography-tandem mass spectrometry (LC-MS/MS) system.

### 2.5. LC-MS/MS Analysis

**Figure 2:**
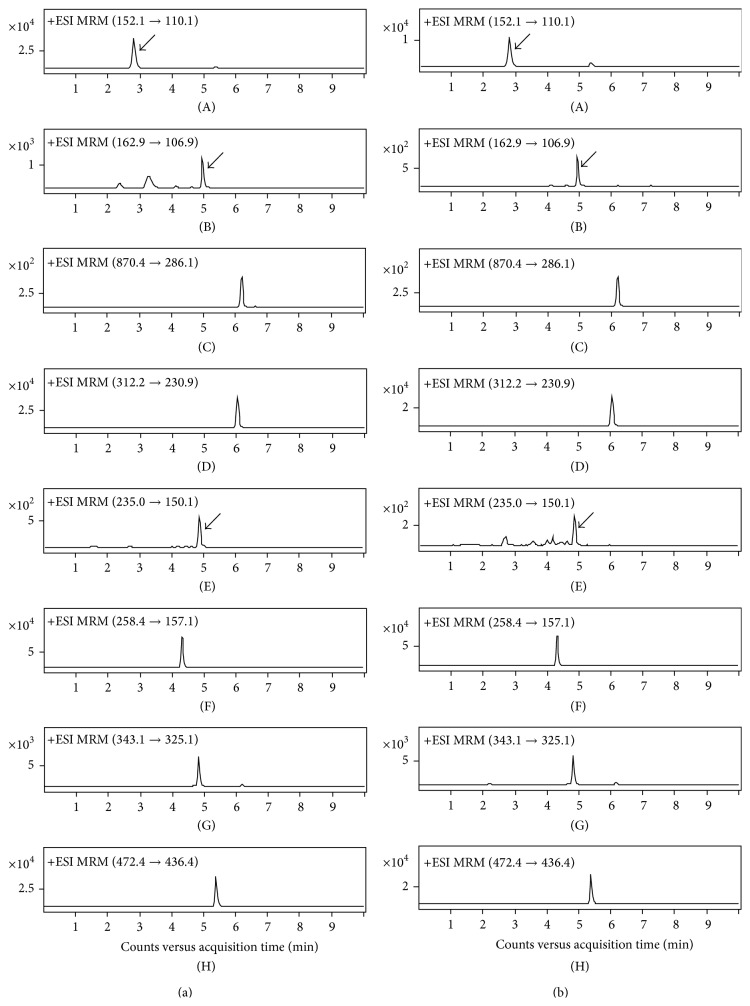
Representative MRM chromatograms of (a) control and (b)* Eurycoma longifolia* extracts-treated human liver microsome samples. Human liver microsomal fraction was incubated with the substrate mixture, NADPH-generating system, and* Eurycoma longifolia* extracts (1000 *μ*g/mL) for 30 min and the formation of the CYP-specific metabolites was determined by LC-MS/MS. (A) Acetaminophen; (B) 7-OH-coumarin; (C) 6-OH-paclitaxel; (D) 4-OH-diclofenac; (E) 4-OH-mephenytoin; (F) dextrorphan; (G) 1-OH-madazolam; (H) terfenadine (IS).

The LC-MS/MS system consisted of an Agilent 1260 series binary pump HPLC system and an Agilent 6460 Triple Quadrupole mass spectrometer (Agilent Technologies, Palo Alto, CA, USA) equipped with an electrospray ionization (ESI) source. Chromatographic separation was achieved using a Fortis C8 column (2.1 mm × 100 mm, 5 *μ*m; Fortis Technologies Ltd., Cheshire, UK). The column temperature was maintained at 40°C. The HPLC mobile phases consisted of 0.1% formic acid in distilled water (A) and 90% acetonitrile in 0.1% formic acid (B). A gradient program was used at a flow rate of 0.2 mL/min; the composition of solvent B was initially set at 15%, gradually increased to 85% over 3 min, and maintained for 1.5 min followed by a reequilibrium for 3.5 min. The mass spectrometer was operated in positive ion mode with multiple reaction monitoring (MRM). The precursor-product ion pairs (Q1/Q3) used in MRM mode are shown in Table 1. The representative MRM chromatograms were shown in Figure 2.

### 2.6. Data Analysis

The half maximal inhibitory concentration (IC_50_) for each CYP isozyme was estimated based on four-parameter logistic nonlinear regression analysis using Sigma Plot version 10.0 (Systat Software Inc., Point Richmond, CA, USA).

## 3. Results and Discussion

**Figure 3:**
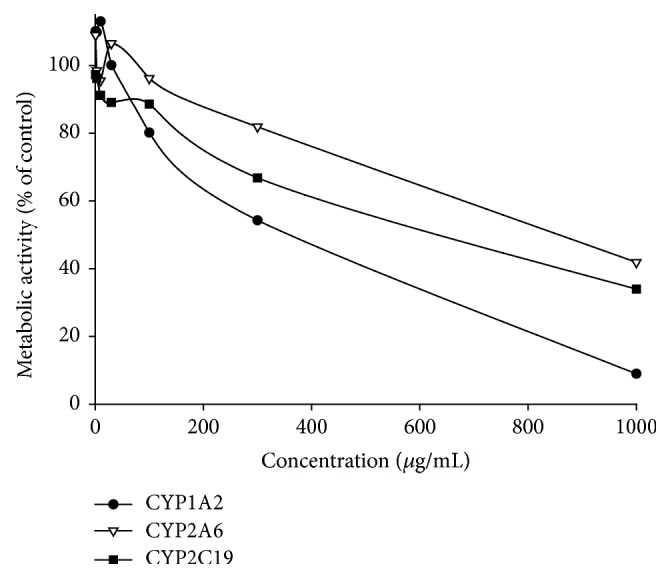
The effects of* Eurycoma longifolia* extracts on metabolic activities of CYP1A2, CYP2A6, and CYP2C19 in recombinant CYP isozymes. Human recombinant CYP isozymes (CYP1A2, CYP2A6, and CYP2C19) were incubated with the corresponding specific substrate, NADPH-generating system, and* Eurycoma longifolia* extracts (- - -) for 30 min and the formation of the CYP-specific metabolite was plotted as the percentage of control.

The inhibitory effects of* E. longifolia* extracts on CYP enzyme activities were investigated in human liver microsomes and recombinant isozymes. The CYP inhibition assay system was tested with well-known CYP selective inhibitors as follows: ketoconazole for CYP3A4, furafylline for CYP1A2, methoxsalen for CYP2A6, quercetin for CYP2C8, sulfaphenazole for CYP2C9, ticlopidine for CYP2C19, and quinidine for CYP2D6. Formation of each CYP-specific metabolite was reduced by >95% following treatment of its corresponding inhibitor, indicating that the assay system was functioning well.* E. longifolia* extracts were tested at concentrations of 1, 3, 10, 30, 100, 300, and 1000 *μ*g/mL for seven CYP isozymes. The results showed that* E. longifolia* extracts inhibited CYP1A2, CYP2A6, and CYP2C19 activities in a concentration-dependent manner. The effects on other isozyme activities were negligible. Subsequently, the inhibitory effects against CYP1A2, CYP2A6, and CYP2C19 isozymes were further evaluated using cDNA-expressed recombinant CYP isozymes.* E. longifolia* extracts showed concentration-dependent inhibition against those enzymes (Figure 3), as observed in the experiments with human liver microsomes (Table 2), with IC_50_ values for CYP1A2, CYP2A6, and CYP2C19 of 324.9, 797.1, and 562.9 *μ*g/mL, respectively. Some reports suggested a classification for CYP inhibitors [14]: potent for IC_50_ values ≤ 10 *μ*M, moderate for IC_50_ values 10–50 *μ*M, and weak for IC_50_ values >50 *μ*M. Based on these criteria, assuming that the average molecular weight of the constituents is 500 da and their average content is approximately 5% in* E. longifolia* extracts, IC_50_ values <500 *μ*g/mL could be of some significance. Thus, possible inhibition of CYP1A2 and CYP2C19 by* E. longifolia* extract should be carefully considered. However, as this implication on some potential for CYP inhibition is based on the* in vitro* data, there is a need to verify this in* in vivo* studies such as interaction studies using animal models or even human subjects.

The major components of* E. longifolia* include quassinoids, *β*-carboline alkaloids, squalene derivatives, and canthinone derivatives [15]. Among them, eurycomanone, a quassinoid, is a principal and characteristic constituent found in* E. longifolia* extracts. The content of eurycomanone in* E. longifolia* root extracts has been reported to 5.65%~9.95% of extracts [16]. As for our extract sample, eurycomanone was found to be the most abundant compound based on the HPLC chromatogram but the content was measured to be lower (1.3%) than the previously reported (shown above) value. This is supposed to result from the differences in extraction method, collection time, cultivated area, and so forth.

With regard to herb-drug interaction via CYP inhibition by the constituents of* E. longifolia* extract, the literature contains one published study of the effect of eurycomanone, a major bioactive compound of* E. longifolia*, on CYP enzyme activities [17]. According to that report, eurycomanone did not potently inhibit CYP isozyme activities and showed IC_50_ values greater than 250 *μ*g/mL. Thus, the authors concluded there was low risk of drug-herb interaction between eurycomanone and CYP drug substrates via CYP inhibition. Therefore, ingredients other than eurycomanone might be involved in the CYP inhibitory effects of* E. longifolia* extracts. However, the literature on CYP-modulating effects of other constituents, such as other quassinoids, *β*-carboline alkaloids, or canthinones, is rather limited. Zhao et al. reported inhibition of CYP3A4 and CYP2D6 enzyme activities by *β*-carboline alkaloids [18]. However, that study only tested those two isozymes; therefore, further studies should be conducted with the major constituents of* E. longifolia* extracts.

Recent studies provide some preclinical or clinical evidence for herb-drug interactions of* E. longifolia* extracts. Salman et al. reported that when propranolol was administered with* E. longifolia*, the *C*_max_ and bioavailability of propranolol decreased by 29% and 42%, respectively, in humans [19]. The authors concluded that this interaction is due to a reduction in absorption rather than increased metabolism of propranolol and advised caution in coadministration. Another paper reported that* E. longifolia* extracts increased the phase I metabolism of rosiglitazone, an antidiabetic drug, in male rat hepatocytes, which suggest the likelihood of CYP enzyme induction by* E. longifolia* extract [20].

*E. longifolia* extract is commonly consumed as a herbal medicine or dietary supplement for its various pharmacological effects. Therefore, it is likely to be coadministered with prescription or conventional drugs, particularly in patients with chronic diseases such as diabetes mellitus, hypertension, and other cardiovascular diseases. Considering the present results and those previously reported in the literature, coadministration of* E. longifolia* products and conventional drugs requires careful attention.

## Figures and Tables

**Table 1:** Precursor-product ion pairs of CYP-specific metabolites for multiple reaction monitoring detection.

P450 isozyme	Metabolites monitored	Precursor ion	Product ion
CYP 1A2	Acetaminophen	152.1	110.1
CYP 2A6	7-OH-coumarin	162.9	106.9
CYP 2C8	6-OH-paclitaxel	870.4	286.1
CYP 2C9	4-OH-diclofenac	312.2	230.9
CYP 2C19	4-OH-mephenytoin	235.0	150.1
CYP 2D6	Dextrorphan	258.3	157.1
CYP 3A4	1-OH-midazolam	343.1	325.1
Internal standard	Terfenadine	472.4	436.4

**Table 2:** Effects of *E. longifolia* extract on CYP-specific metabolite formation in human liver microsomes.

P450 isozyme (Specific metabolite)	Metabolite formation (% of control)						
	*E. longifolia* extract (*µ*g/mL)						
	1	3	10	30	100	300	1000
CYP1A2 (Acetaminophen)	92.0	95.5	91.0	89.1	71.3	68.4	26.9
CYP2A6 (7-OH-coumarin)	96.1	104.1	79.8	86.8	79.1	76.6	56.4
CYP2C8 (6-OH-paclitaxel)	88.1	95.6	85.5	100.1	86.1	86.7	103.3
CYP2C9 (4-OH-diclofenac)	92.2	104.0	93.6	97.2	92.3	84.9	88.0
CYP2C19 (4-OH-mephenytoin)	91.3	106.6	85.7	90.1	77.8	50.2	45.9
CYP2D6 (Dextrorphan)	91.1	88.8	81.4	93.3	82.6	80.0	77.4
CYP3A4 (1-OH-midazolam)	96.7	106.4	88.3	93.6	90.0	97.7	101.4
